# Spatiotemporal Coordination of Rac1 and Cdc42 at the Whole Cell Level during Cell Ruffling

**DOI:** 10.3390/cells12121638

**Published:** 2023-06-15

**Authors:** Siarhei Hladyshau, Jorik P. Stoop, Kosei Kamada, Shuyi Nie, Denis Tsygankov

**Affiliations:** 1 School of Biology, Georgia Institute of Technology, Atlanta, GA 30332, USA; 2 Wallace H. Coulter Department of Biomedical Engineering, Georgia Institute of Technology and Emory University, Atlanta, GA 30332, USA; 3 Faculty of Medicine, The University of Tokyo, Tokyo 113-8654, Japan

**Keywords:** multiscale modeling, morphodynamics, Rho family GTPases, FRET-based biosensors, cytoskeletal regulation

## Abstract

Rho-GTPases are central regulators within a complex signaling network that controls cytoskeletal organization and cell movement. The network includes multiple GTPases, such as the most studied Rac1, Cdc42, and RhoA, along with their numerous effectors that provide mutual regulation through feedback loops. Here we investigate the temporal and spatial relationship between Rac1 and Cdc42 during membrane ruffling, using a simulation model that couples GTPase signaling with cell morphodynamics and captures the GTPase behavior observed with FRET-based biosensors. We show that membrane velocity is regulated by the kinetic rate of GTPase activation rather than the concentration of active GTPase. Our model captures both uniform and polarized ruffling. We also show that cell-type specific time delays between Rac1 and Cdc42 activation can be reproduced with a single signaling motif, in which the delay is controlled by feedback from Cdc42 to Rac1. The resolution of our simulation output matches those of time-lapsed recordings of cell dynamics and GTPase activity. Our data-driven modeling approach allows us to validate simulation results with quantitative precision using the same pipeline for the analysis of simulated and experimental data.

## 1. Introduction

Cell motion and changes in shape (also referred to as morphodynamics) are processes driven by the complex, multiscale machinery of the cytoskeleton. The central regulators of this machinery are small GTPases that control both membrane protrusion and retraction [1,2,3,4]. GTPases control protrusion by activating F-actin polymerization through nucleation-promoting factors (downstream of GTPases Rac1 and Cdc42) [5], and they modulate retraction by controlling actomyosin contractility through Rho kinase (downstream of GTPase RhoA) [2]. Rac1 and Cdc42 induce local activation of formins (mDia1 and mDia2) and Arp2/3, which leads to actin polymerization and membrane protrusion driven by the resulting F-actin mesh [6]. Over the last decades, numerous studies have shed light on the mechanisms of RhoA, Rac1, and Cdc42 signaling (reviewed in [3,7,8,9,10,11]). Many regulators of GTPase signaling were discovered, including guanine nucleotide exchange factors (GEFs) that control GTPase activation by catalyzing the exchange of GDP with GTP, and guanine activating proteins (GAPs) that control GTPase deactivation by catalyzing the GTP hydrolysis [8]. Some of these proteins were shown to interact with several GTPases or have dual GEF/GAP functions [10,12,13,14]. Such multi-target regulators may work as a crosslink between Rac1 and Cdc42. Indeed, it was reported that induced activation of Cdc42 can lead to the activation of Rac1, but the specific regulators and the details of this mechanism are not fully understood [15]. Similarly, the crosstalk of small GTPases with other signaling pathways is a matter of intensive research [16]. It was reported that signaling pathways related to cytoskeleton regulation, including the Rac1 pathway, operate downstream of an excitable signal transduction network that involves Ras and PI3K pathways [17,18]. Given that many effectors of small GTPases have a pleckstrin homology (PH) domain, which binds to phosphatidylinositol lipids, Rac1 and Ras/PI3K signaling networks can be coupled in multiple ways (e.g., through interactions of Rac1 GEFs with PIP3) [16]. Additionally, crosstalk between phosphoinositides and Cdc42 was reported [19]. Despite the growing knowledge of the roles of various cytoskeletal regulators, there is still no holistic understanding of the intertwined small-GTPase signaling pathways. This lack of understanding is especially problematic in the context of their coordinated activity during cell motion and interactions with the extracellular environment.

The application of fluorescence resonance energy transfer (FRET) biosensors has facilitated the study of small GTPases and their effectors in live cells with high spatial and temporal resolution [20]. In particular, this technology has allowed researchers to investigate the quantitative relationship between the proteins’ activity and the velocity of membrane protrusion [20,21]. Several studies showed that during protrusion, the velocity peak precedes the peak of Rac1 and Cdc42 activity [22,23]. This timing appears to be counterintuitive as the F-actin polymerization that drives protrusion is regulated downstream of these GTPases. As a possible explanation, Yamao et al. showed that the response function between biosensor activity and membrane velocity displays the properties of a differentiator circuit [24]. The authors specifically suggested that the membrane dynamics is regulated by the temporal derivative of active forms of Rac1 and Cdc42, which may explain the time shift between membrane velocity and GTPase activity. Yet, the mechanism that would explain how cell membrane protrusion could be regulated by the temporal derivative of GTPase activity has not been described. Using cross-correlation analysis, Marston et al. showed that in the epithelial breast cancer cell line (MDA-MB-231), there is no measurable time shift between the peaks of Cdc42 and Rac1 activities [20]. On the other hand, some studies reported that Rac1 activation could be induced by Cdc42 [7,15] and that timing between the activation of two GTPases may vary [25,26]. These results suggest that the relative timing and regulation of GTPase may be cell-type specific and depend on the biological context or type of regulation. Partial correlation analysis also revealed that the guanine nucleotide exchange factor (GEF) Asef contributes to the regulation of cell protrusion by both Cdc42 and Rac1 and that the degree of the GEF’s influence on the two GTPase pathways is different [20]. This computational analysis takes advantage of the simultaneous visualization of two GTPases or one GTPase and its regulator and provides valuable insights regarding mutual influences between signaling components. However, correlation analysis does not provide the full mechanistic picture of a regulatory process. To shed light on this situation, here we propose a computational model that couples GTPase signaling and cell edge motion. We quantitatively analyze the relationships of GTPase activity level and its rate of change with cell edge position and velocity, which allows us to compare the experimental data and our simulation results directly.

The two key features characteristic of models used in the theory of biological morphogenesis are autocatalytic activation of the pattern-forming component and a significant difference in the diffusion coefficient of an activator and an inhibitor [27,28,29,30]. This theory was originally proposed by Alan Turing [31] and further developed by Hans Meinhardt [27,32,33]. Subsequently, researchers applied these developments to model small GTPases under the assumption of an underlying activator-inhibitor mechanism that allows the representation of their localized activation and wave dynamics [34,35]. Indeed, GTPases exhibit the properties of autocatalytic activation through positive feedback [36,37,38,39] and a significant difference in the diffusion coefficients of active and inactive forms, which are due to interactions of active GTPases with the membrane [14,40]. While activator-inhibitor models are consistent with the observed features of GTPase activity in cells, it is hard to relate the model parameters to the biochemical characteristics of the components in the signaling motif [17,41]. Recent studies proposed more detailed models to improve the interpretability of the earlier modeling results [12]. Specifically, to account for the switch of small GTPases between active and inactive states, the mass-conserved reaction-diffusion model (MCRD, also known as the wave-pinning mechanism) was proposed as a framework to study the quantitative properties of GTPase signaling [30,42]. MCRD was successfully applied to study cell polarization [30,43,44,45,46]. Importantly, the model captures a Turing-unstable type of behavior, in which the activity of small-GTPases remains non-homogeneous, as well as the transition to the excitable regime, in which a stimulus-induced activation is required to generate GTPase patterning. Therefore, the MCRD model can be used to represent a core signaling motif of GTPases and, when coupled with downstream and upstream effectors, can reproduce rich dynamics, including the formation of complex cortical waves of GTPase activity [47]. A more detailed variation of this model was also successfully applied to study the coordination of RhoA and Rac1 in the leading and trailing edges of moving cells [48]. A similar modeling method was applied to analyze drug resistance to cancer treatment [49]. Thus, the MCRD-based modeling framework is emerging as a powerful quantitative approach to studying the mechanisms of coordinated spatiotemporal behavior of small-GTPases and their effects on cell morphodynamics.

In this work, we investigate the regulation of Rac1 and Cdc42 in the epithelial breast cancer cell line (MDA-MB-231) during cell membrane ruffling, a phenomenon that is characterized by the formation and retraction of actin-rich membrane protrusions at the periphery of a spread cell [50]. We do not consider the formation of membrane folds and their detachment from the surface, also called membrane ruffing in literature [51]. For the development of the model, we used experimental data from a study on Rac1 and Cdc42 regulation with multiplexed FRET biosensors [20]. In experiments of this type, cell morphodynamics typically involves two timescales: fast localized oscillations of the cell outline and a slow change of the overall cell shape. To assess these features of the experimental data and provide accurate measurements of the protrusive activity, we developed an automated image analysis pipeline. This pipeline tracks fast cell edge movements and local biosensor signals along many evenly distributed line segments, which slowly move together with the overall cell shape.

To understand the regulatory mechanism of these dynamics, we developed a computational framework to model cell morphodynamics coupled with a reaction-diffusion representation of GTPase activity in the cell. Using this framework, we showed that the regulation of cell edge velocity by kinetic rates of GTPase activation accurately reproduces the relationship between the protrusive activity and the biosensor signal in experimental data, consistent with the interpretation of this relationship as a differentiator circuit.

Using our image analysis pipeline, we also analyzed the mouse-embryonic fibroblasts (MEFs) data, published previously by MacNevin et al. [52], and observed a delayed activation of Rac1 relatively Cdc42. Although we obtained a delay of 5 s, which is at the limit of the temporal resolution of the available imaging data, we cannot exclude that such a delay is a true feature of the cell edge motion in MEFs. An experiment with a higher frame rate is needed to confirm the delay and provide a more reliable and accurate measurement of its value. In the meanwhile, we sought to investigate coupled models of Rac1 and Cdc42 regulation that could quantitatively reproduce experimentally observed dynamics and proposed alternative signaling models that can explain the simultaneous (in the case of breast cancer cells) or delayed (in the case of MEFs) activation of both GTPases.

In one of our considered models, Rac1 and Cdc42 form a bidirectionally coupled system, where Cdc42 activity forms a polarized pattern that defines regions of high protrusive activity, while Rac1 activity drives the protrusion-retraction cycle with feedback to Cdc42. In an alternative model, both Rac1 and Cdc42 respond to the same upstream regulator. We hypothesize that such a regulator can work through the PI3K signaling pathway and activate Rac1 and Cdc42 activity through phosphoinositide regulation. Our final model proposes the presence of a common upstream regulator, with crosstalk between two GTPases in the form of positive feedback from Cdc42 to Rac1. We show that such crosstalk could synchronize Cdc42 and Rac1 dynamics and compensate for the delayed activation.

## 2. Results

### 2.1. Coupling Reaction-Diffusion Models of GTPase Regulation and Cell Morphodynamics

To investigate the mechanisms of cell membrane ruffling, we developed a morphodynamic model that allows us to represent dynamically changing cell shapes with a resolution directly matching experimental imaging data. Integrated with a reaction-diffusion system of equations describing Rho-GTPase signaling, the morphodynamic model captures spatial and temporal properties of the experimentally observed GTPase activity. For quantitative comparisons of our results with observations, we developed an image analysis pipeline to extract cell edge velocity and near-edge GTPase activity in both experimental and simulation data. The following sections describe three different aspects of our computational approach, as well as the proposed data pipeline.

#### 2.1.1. Modeling Cell Morphodynamics

**Figure 1:**
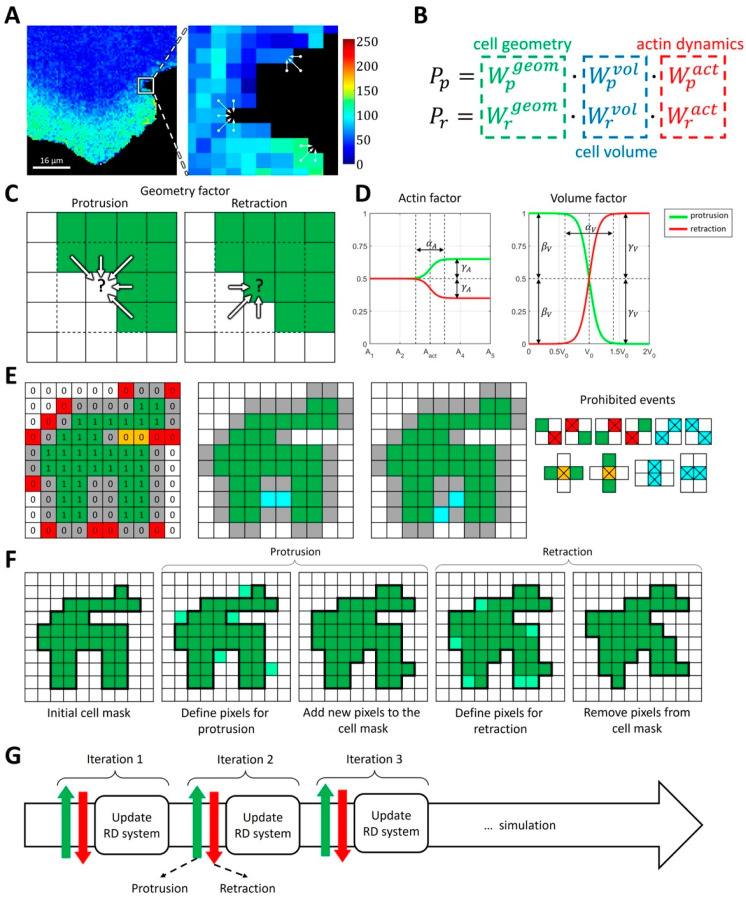
Concepts of the proposed integrative model of cell morphodynamics. (**A**) A cell is represented as a binary 4-connected object on a square grid. Changes in cell shape are represented as a series of protrusion/retraction events. Colormap indicates FRET biosensor signal for active Rac1. (**B**) The probabilities of protrusion and retraction depend on three factors: geometry factor (green), volume factor (blue) and actin factor (red). (**C**) The geometry factor is defined for each pixel at the outline of the cell and depends on the values of cell mask (green) in the cell’s neighborhood. Convex regions of the cell mask with high curvature are less likely to protrude and more likely to retract. Concave regions of the cell mask with negative curvature are more likely to protrude and less likely to retract. (**D**) Actin and volume factors have the form of sigmoid functions and represent deviations from the mid-value value of 0.5, depending on the regulator of actin or cell volume. Parameters include the threshold of activation ($A_{act}$, $V_{0}$), sharpness of the response ($\alpha_{A}$, $\alpha_{V}$), deviation from 0.5 for increased regulation ($\gamma_{A}$, $\gamma_{V}$), and decreased regulation ($\beta_{V}$). (**E**) Protrusion events take place at the outline (gray 0 s) of the cell mask (green 1 s). To maintain the unity of simulation domain we prohibit diagonal events (red 0 s). Events that lead to the formation of bubbles are also prohibited (orange 0 s). Simultaneous updates of the pixels at a cell outline can lead to the formation of bubbles or diagonal events (blue). Such events are processed separately so that only one of them takes place. During retraction the logic is reversed: 0 s represent cell mask and 1 s represent empty space. (**F**) During cell shape updates, each protrusion event is followed by retraction event. (**G**) Cell dynamics is represented as an iterative process where the protrusion-retraction cycle is followed by the update of intracellular signaling (reaction-diffusion, RD model).

We built a model of a moving cell as a sequence of stochastic protrusion-retraction events. The probability of each such event is modulated by GTPase Rac1 activity through actin polymerization. For direct comparisons of the model output with experimental microscopy data, we performed the simulation on a square grid with the resolution directly matching the imaging data. Each cell of an arbitrary shape is represented as a binary object on a square grid (also referred to as the cell mask M). The cell shape is updated stochastically by adding pixels (protrusion) and removing pixels (retraction) at the cell edge (Figure 1A). Conceptually, such an approach is similar to the Cellular Potts Model (CPM) [53]. However, our implementation has some major differences. Importantly, we separate protrusion and retraction events, which allows us to distinguish between the separate regulation mechanisms of these two processes and model them differently if needed. We also do not assume a global Hamiltonian to define the probabilities of cell shape changes because such a formulation is somewhat abstract and cannot be easily related to cell morphodynamics. Instead, we consider it very beneficial if our model can directly be related to biochemical regulation and interpreted from a biomechanical perspective.

Taking these considerations into account, we define the probabilities of protrusion and retraction with three contributing factors: local geometry (local curvature of the cell membrane), overall time-dependent cell volume (change in the size of the simulation domain), and local actin polymerization (controlled by the activity of GTPases in the RD model) (Figure 1B). Specifically, we define

$$\mathrm{Protrusion}:\;P_{p}=W_{p}^{geom}\cdot W_{p}^{vol}\cdot W_{p}^{act}$$

$$\mathrm{Retraction}:\;P_{r}=W_{r}^{geom}\cdot W_{r}^{vol}\cdot W_{r}^{act}$$

For each of these factors, we parametrize the probability of protrusion and retraction so that they contribute to the membrane velocity based on biological and physical rationale. The geometry factor describes the response to local membrane curvature (Figure 1C) so that convex regions of the cell mask with high positive curvature are less likely to protrude and more likely to retract. In contrast, concave regions of the cell mask are more likely to protrude and less likely to retract. This setup is well suited for modeling broad lamellipodia-like protrusions, as they occur during cell ruffling. For modeling filopodia-like protrusions or other high-curvature cell shapes, the setup would need to be modified accordingly. The volume factor accounts for the effects of cell size changes, which may happen on a larger time scale than a single protrusion/retraction event. In our implementation, the increase in cell size with respect to the initial size leads to decreased protrusion and increased retraction probabilities and vice versa (Figure 1D). As a result, cell size remains close to the initial value, but with slight stochastic deviations. The extent of such deviations can be regulated by the parameters of the model (see Materials and Methods).

Finally, the actin factor describes the regulation of cell morphodynamics by GTPase signaling pathways that induce actin polymerization driving cell edge movement. We assume that, in the absence of GTPase activity, the regulation is neutral, i.e., that this factor does not contribute to the relative change in the probabilities of protrusion and retraction (Figure 1D). In other words, when the concentration of the active form of GTPase is below the specified threshold, the actin factor is equal to 0.5 for both the protrusion and retraction probabilities. The upregulation of GTPase activity leads to an increased protrusion rate and decreased retraction rate. In such a setup, the cell protrusion (when GTPase is locally activated) is followed by a relaxation phase (when GTPase is deactivated), where the cell membrane retracts based on the other probability factors. The functional forms of each factor are described in the Methods section.

Based on the different probabilities, the overall dynamics of cell shape are calculated as a sequence of protrusion/retraction events using the Monte Carlo algorithm (Figure 1E–G). During the protrusion step, new pixels are added to the cell edge (i.e., some of the background pixels at the very edge of the cell mask become foreground pixels). During the retraction step, pixels are removed from the cell edge (i.e., some of the pixels forming the cell outline become background pixels). To maintain the 4-connectedness, unity of the cell mask, and the absence of holes, our algorithm automatically prohibits pixel addition/removal that would lead to such distortions.

Overall, this model represents cell motion as a sequence of stochastic fluctuations of the cell membrane, modulated by the effects of changes in cell volume, membrane curvature, and the regulation of actin polymerization with intracellular signaling.

#### 2.1.2. Spatiotemporal Model of GTPase Activity during Cell Ruffling

**Figure 2:**
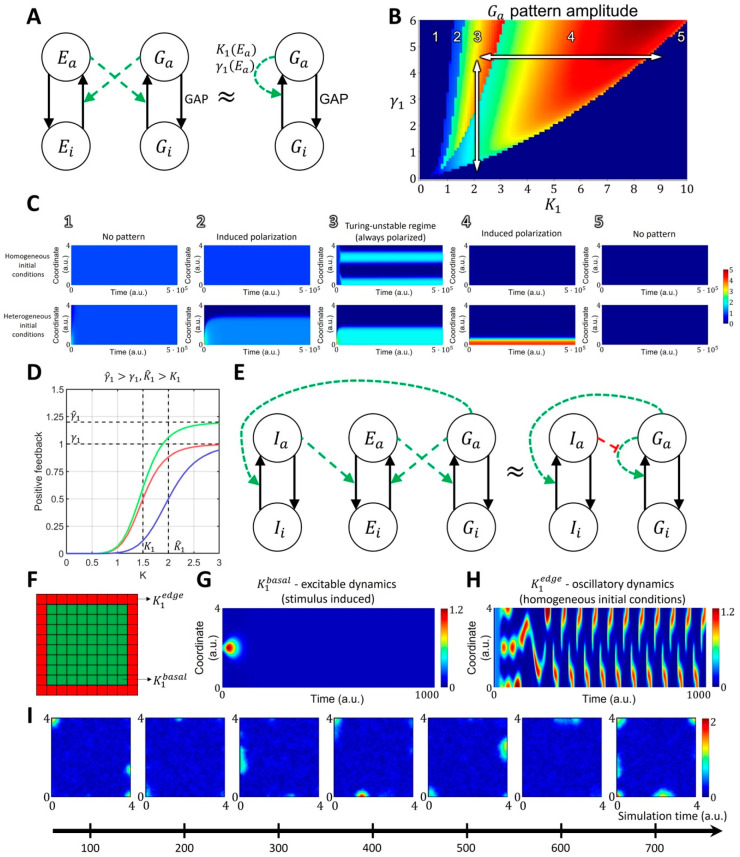
Reaction-diffusion model of GTPase activity in cell membrane ruffling. (**A**) The two-component MCRD model of GTPase activity ($G_{a}$—active GTPase, $G_{i}$—inactive GTPase) can be interpreted as an approximation of an extended four-component model, where active GTPase increases the activation rate of GEF ($E_{a}$—active GEF, $G_{i}$—inactive GEF), and active GEF, in turn, increases the activation rate of GTPase. (**B**,**C**) In the phase space of $K_{1}$ and $\gamma_{1}$ parameters, the MCRD model can operate in several distinct regimes: 1. No pattern (stable, homogeneous, high-activity state, stimulus cannot induce polarization); 2. Stimulus-induced deactivation (stable, homogeneous, high-activity state, stimulus can induce polarization); 3. Turing-unstable regime (unstable homogeneous state, stimulus is not required to induce polarization, as it can be initiated by any small perturbation); 4. Stimulus-induced activation (stable, homogeneous, low-activity state, stimulus can induce polarization) and 5. No pattern (stable, homogeneous, low-activity state, stimulus cannot induce polarization). The white arrows in B represent the transition of the two-component system between different parts of the phase space in response to the changes in parameters $K_{1}$ (the rate of GEF deactivation) and $\gamma_{1}$ (the concentration of GEF regulators). The activity patch of Rho-GTPases, formed in the Turing-unstable regime 3, can be turned off if the system moves to an inactive monostable regime 5 by either increasing $K_{1}$ or decreasing $\gamma_{1}$. (**D**) The regulation of positive feedback in the MCRD changes the shape of the Hill function. An inhibitor can increase $\gamma_{1}$ parameter (magnitude of positive feedback, green curve) or increase $K_{1}$ parameter (threshold of positive feedback activation, blue curve). (**E**) Signaling motif where active GTPase G activates inhibitor I, which in turn increases the rate of GEF deactivation. In the simplified model containing the MCRD motif, it is equivalent to the increase in $K_{1}$ parameter. Green and red colors represent positive and negative regulation, respectively. (**F**) In the 2D model, we assumed an increased GTPase activity at the boundary of the simulation domain (decreased $K_{1}^{edge}$ for red pixels in comparison to $K_{1}^{basal}$ for green pixels), which corresponds to increased density of GEF proteins with a curvature-sensitive domain. (**G**) $K_{1}^{edge}$ corresponds to the oscillatory dynamics. (**H**) $K_{1}^{basal}$ corresponds to the excitable dynamics. (**I**) The GTPase dynamics (shown as snapshots from the timeseries) in the 2D model show the formation of transient activity patches at the boundary of the simulation domain which corresponds to the experimental observation when GTPase (Rac1 and Cdc42) are activated at the cell edge.

FRET biosensor data [20,21] show that during cell membrane ruffling, Rac1 and Cdc42 activities correlate with membrane velocity and form transient localized patches of activity close to the cell edge. To represent such dynamics in simulations, we used an MCRD model with autocatalytic feedback as a core signaling motif (see Methods). Such a model can be interpreted as a coarse-grained approximation of a more complex signaling motif with positive feedback through the activation of GTPase effectors (e.g., GEFs), which in turn increases the activation of GTPase (Figure 2A).

Mathematically, converting the extended model into the coarse-grained one (see Supplementary Text S1) leads to a dependence of parameter $\gamma_{1}$ (the maximum of the resulting Hill function) on the total concentration of GTPase effectors, such as GEF, and dependence of parameter $K_{1}$ (the threshold of activation) on the rate of effector deactivation. Depending on its parameters, the MCRD model can operate in the excitable regime or in the Turing-unstable regime. In the excitable regime, a finite stimulus is needed to induce the formation of an activation patch, whereas the homogeneous state is unstable in the Turing-unstable regime, and no stimulus is needed to induce GTPase activation (Figure 2B,C). Outside of these regimes, the system is in an inactive state where no patterns are formed with or without a stimulus.

When an activity patch is formed in either the excitable or Turing-unstable regime, it can be deactivated by a regulator that moves the system to the inactive regime. We visualized this behavior with the phase space portrait for parameters $\gamma_{1}$ and $K_{1}$ (Figure 2B,C). Thus, in order to model a system with the transient formation of an activity patch, we coupled the core signaling motif and a regulator that modulates the positive feedback in the system (e.g., negatively regulates the activation of GEFs). This way, active GTPase increases the activation of a regulator that deactivates the GTPase by increasing the threshold of the autocatalytic activation ($K_{1}$ parameter, Figure 2D,E).

This coupled system can be excitable or oscillatory. In the excitable state, the activity is induced by a stimulus and decays due to the negative feedback from the regulator, and in the oscillatory state, the core motif switches between Turing-unstable and inactive states due to the negative feedback from the regulator (Figure 2F–H). Previous experiments had shown that there is a narrow band right at the cell edge where GTPase GEF Asef remains active as the cell ruffles, while in the other areas, GEF activity varies during the protrusion/retraction cycle [54]. Such persistent GEF activation at the very edge of the cell may reflect the fact that many GTPase effectors contain the curvature-sensing BAR domain [15,54,55,56,57]. Thus, for the 2D implementation of our model, we assumed that the activity of GTPases is slightly higher at the boundary of the simulation domain than everywhere else. To implement this condition in the model and achieve the spontaneous formation of activity patches as observed in cell ruffling, we chose the parameter $K_{1}=1.4$ at the one-pixel-wide boundary of the simulation domain and $K_{1}=2.1$ a.u. elsewhere (Figure 2F). In our implementation at the boundary of the simulation domain, the system operates in the oscillatory regime, while inside the simulation domain, it is in the excitable state (Figure 2G,H). This setup reproduces localized and transient activity of GTPases at the edge of the simulation domain, matching the experimentally observed dynamics during cell ruffling (Figure 2I).

#### 2.1.3. A Pipeline for the Analysis of Cell Edge Velocity and GTPase Activity in Experiments and Simulations

**Figure 3:**
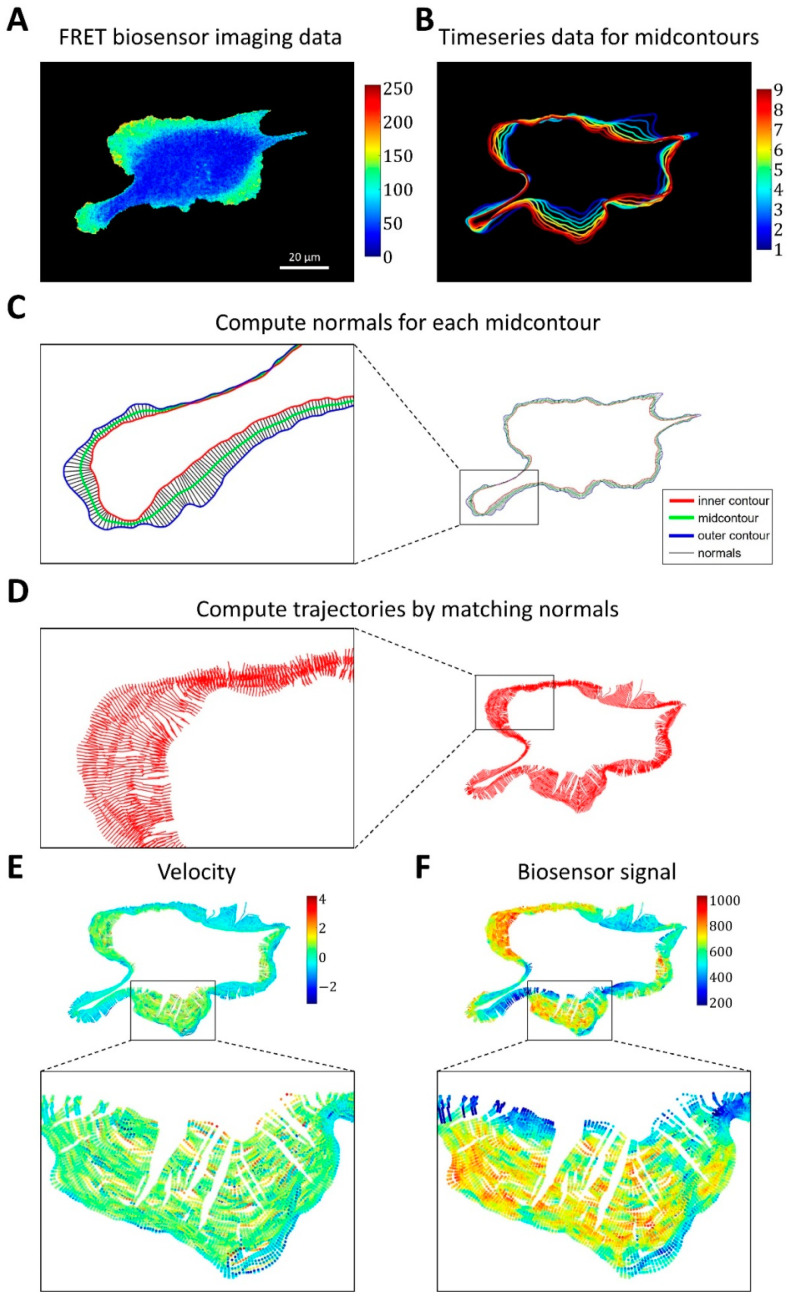
Image analysis pipeline for simultaneous analysis of cell edge velocity and biosensor signal. (**A**) The input of the pipeline consists of time series of FRET biosensor data in cells. (**B**) The time-series data were split into time intervals of 20 frames, and for each time interval a mid-contour was computed. (**C**) For each vertex of the computed mid-contour, the perpendicular lines were found and their intersections with the inner and outer contours defined the normals (i.e., segments between the inner and outer contours perpendicular to the mid-contour). (**D**) Cell membrane trajectories were computed by matching the closest normals. (**E**,**F**) Cell edge velocity and biosensor signal were tracked along the computed trajectories at the points of intersection with the cell edge.

To facilitate direct comparisons between simulation results and experimental observations, we developed an image analysis pipeline allowing simultaneous analysis of cell edge velocity and biosensor signal during cell ruffling. This pipeline was motivated by the need to account for two timescales of cell movement: fast cell edge fluctuations (ruffling) and a slow change of the overall cell shape. To this end, we first processed observed time-series data (Figure 3A) by extracting cell masks (see Methods) and splitting the whole time series into 20-frame intervals. For each time interval, we identified the outer contour, which represents the smallest region that encloses all cell masks in the time interval (i.e., the union of cell masks), and the inner contour, which represents the overlap of all cell masks in the time interval (i.e., the intersection of cell masks). The discretized representation of the contours was resampled with a 1-pixel distance between the consecutive contour points.

Based on outer and inner contours, we used an iterative algorithm that converges to a mid-contour between the outer and inner ones. First, the algorithm computes normal lines for each point of the outer contour, finds their intersections with the inner contour, and sets the midpoints of these lines between the outer and inner contours as Contour 1. Next, the algorithm computes normal lines at each point of the inner contour, finds their intersections with the outer contour, and sets the midpoints as Contour 2. These two steps are repeated in subsequent iterations but with Contour 1 and Contour 2 instead of the outer and inner contours. The iterative process is interrupted when the average distance between the two new Contours 1 and 2 becomes smaller than a pre-defined tolerance (0.1 pixels, see Methods). Once the mid-contours are computed for each 20-frame time interval (Figure 3B), we record the normal lines to these mid-contours and the intersection of each cell outline in this interval with the corresponding lines (Figure 3C). Finally, we match the normal lines from consecutive time intervals based on the proximity of the last set of intersections in one interval and the first set of intersections in the next one. In this manner, we constructed piecewise linear trajectories, along which the points of the cell outline as they move over time (Figure 3D).

For each point of each trajectory, we computed the velocity (based on position change along the trajectory) and biosensor signal (by averaging the values in the neighborhoods of the points) (Figure 3E,F). Using this method, we represent cell velocity and biosensor signal as kymographs, with the x-axis showing the numerical index of the trajectories and the y-axis representing time.

**Figure 4:**
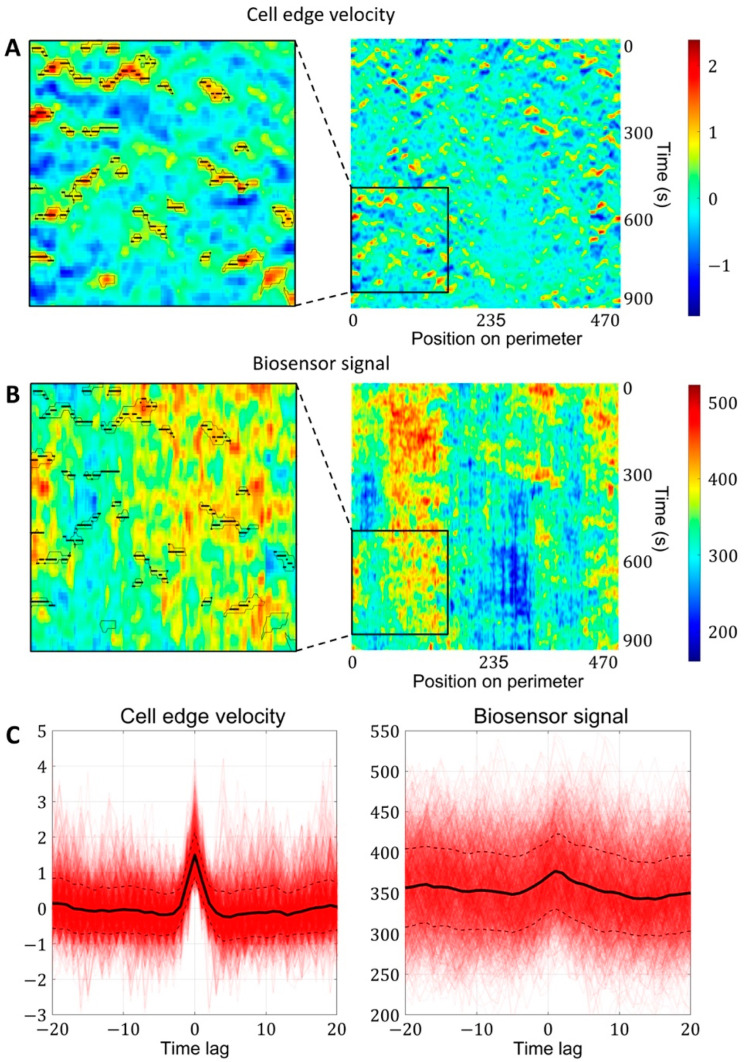
Simultaneous analysis of cell edge velocity and biosensor signal in the neighborhood of local maxima of cell edge velocity. (**A**) In the resulting velocity kymographs, we defined the areas of increased velocity values (black outlines) and found the points of local maxima within these regions (black dots). (**B**) The points of local maxima were transferred from the velocity kymograph to the kymograph with biosensor signal kymograph. (**C**) Both for velocity and biosensor signal, the values in the neighborhood of the identified points were extracted from kymographs and averaged to obtain the relative temporal comparison.

Finally, we used these kymographs to analyze the relationship between GTPase activity and the dynamics of cell edge motion near the time and location of the velocity peaks (see Methods, Figure 4A–C).

Taken together, this method provides a combined quantification of cell edge velocity and biosensor signal (Rho-GTPase activity) both in experimental data and in simulations, which we can calibrate to match the resolution of the imaging data.

### 2.2. Cell Edge Velocity Is Regulated by the GTPase’s Rate of Activation Rather than Its Concentration Value

**Figure 5:**
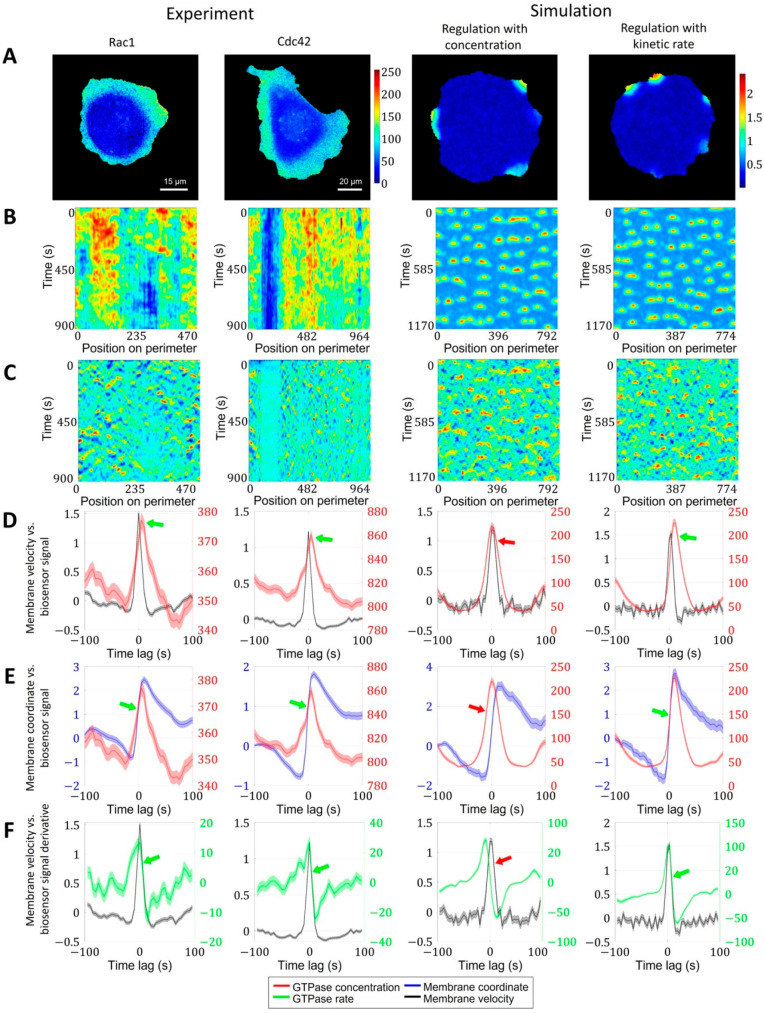
Regulation of GTPase activation by GTPase activation rate. We performed the same analysis for Rac1 and Cdc42 data in a breast cancer cell line and in two simulation setups, where actin factor in the model is regulated by the absolute value of the GTPase concentration and by the kinetic rate of GTPase activation. (**A**) Snapshots of GTPase activity (colormaps represent FRET biosensor signal in experimental images and active GTPase concentration in simulations). (**B**) Kymographs of GTPase activity. (**C**) Kymographs of cell edge velocity. (**D**) Comparison of cell edge velocity and biosensor signal (GTPase concentration). (**E**) Comparison of cell edge coordinate and biosensor signal (GTPase concentration). (**F**) Comparison of cell edge velocity and temporal derivative of biosensor signal (GTPase concentration). Features that are inconsistent with the experiment are marked with red arrows. Green arrows indicate agreement with the experiment.

Using our proposed image analysis pipeline, we analyzed the dynamics of the cell membrane and GTPase activation in experimental data from breast cancer cells (MDA-MB-231). Both for Cdc42 and Rac1, the peaks of activity followed the peak of membrane velocity (Figure 5A–D, left two columns). Given that actin polymerization and membrane protrusion are regulated downstream of Rac1 and Cdc42, such results may look counterintuitive. However, this effect was reported in several studies [20,21,24]. Yamao et al. suggested that the response function of a biosensor signal representing the regulation of membrane velocity has the properties of a differentiator circuit [24]. Unfortunately, the mechanism of such a regulatory mechanism is still not understood.

To provide insight into this phenomenon, we first analyzed the dependence of membrane position (rather than velocity) on the biosensor signal and also the dependence of membrane velocity on the temporal derivative (i.e., the rate of change) of the biosensor signal. During protrusion, the change in membrane position occurs simultaneously with the increase in the biosensor signal (Figure 5E). The membrane velocity decreased simultaneously with the temporal derivative of the biosensor signal (Figure 5F). The time shift of Rac1 and Cdc42 peaks relative to the velocity peak was identical (each ~5 s), which implies simultaneous activation of both GTPases consistently with the previous report [20].

Next, we sought the simplest model that reproduces the temporal properties of Rac1 and Cdc42 activation in our 2D simulations of cell morphodynamics. We applied the same image analysis pipeline to the output (time-lapse images) of the pertinent models. For the first model setup (see Methods, Equations (11)–(14)), we assumed the actin factor in the model to be regulated by the concentration values of the active form of GTPases (Figure 5, third column). In this case, the peak of velocity was closely aligned with the peak of GTPase concentration, the increase in the membrane position along trajectories followed the GTPase concentration peak, and the increase in the temporal derivative of the concentration preceded the membrane velocity increase (Figure 5D–F, red arrows). Thus, none of the three quantitative characteristics agree with the experimental data.

As an alternative model setup, we made the regulation of the actin factor dependent on the temporal derivative of GTPase activation (Figure 5, rightmost column). In contrast to the first setup, this design reproduced the temporal shift of GTPase activity relative to the velocity peak, as well as the simultaneous increase in the membrane position with the GTPase concentration and the simultaneous decrease in the membrane velocity with the decrease in the temporal derivative of the GTPase concentration (Figure 5D–F, green arrows). These results suggest that membrane velocity is regulated by the kinetic rate of GTPase activation rather than the concentration of active GTPase. We interpret this finding in the following way: to power membrane protrusion during cell ruffling, it is not sufficient to maintain a certain level of GTPase activity. Instead, the membrane continues to protrude as long as the GTPase activity continues to increase. Once a max level of GTPase activity is reached, the protrusion stalls and the retraction cycle are initiated.

### 2.3. Cell-Type Specific Relationship between Peaks of Rac1 and Cdc42 Activity Can Be Reproduced with a Unified Model Operating in Different Dynamic Regimes

**Figure 6:**
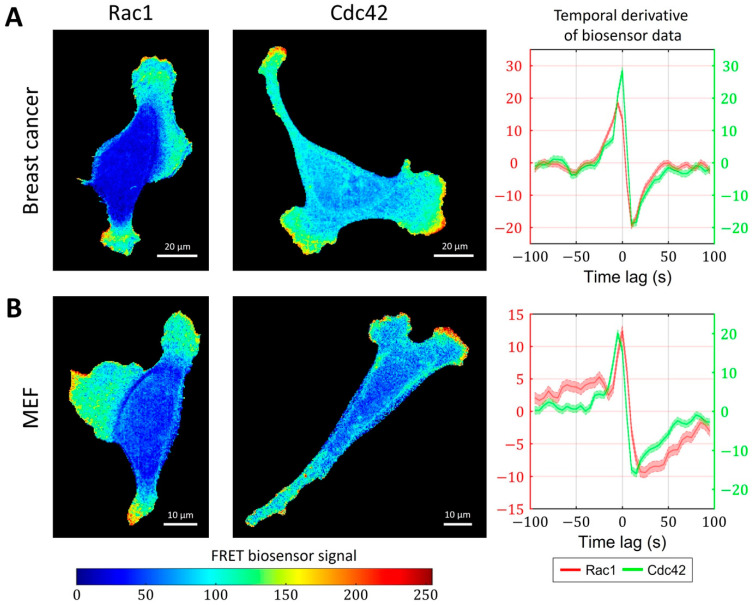
Simultaneous and delayed activation of Rac1 and Cdc42. (**A**) Breast cancer cell line shows coherent activation of Rac1 and Cdc42. (**B**) A delay between Cdc42 and Rac1 is observed in an MEF cell line.

So far, we have focused on the analysis and modeling of GTPases activity during the ruffling of breast cancer cells (Figure 6A). In this cell type, the peaks of Rac1 and Cdc42 activity occur simultaneously (i.e., with zero time delay). However, in mouse embryonic fibroblast (MEF) cells, FRET biosensor data [52] showed a small but distinctive time lag between the activation of the two GTPases. Peaks of Cdc42 activity precede peaks of Rac1 activity by 5 s on average (Figure 6B). To understand the temporal regulation of GTPase activity, we considered several minimal models and explored the possibility to reproduce different time delays between Rac1 and Cdc42. We assumed that the time delay could be controlled by modulating the parameters involved in the feedback loops between the signaling components and by the sensitivity of Rac1 and Cdc42 to the upstream effector.

We considered models with ‘crosstalk’ between Rac1 and Cdc42, as was previously reported in several studies [10,15,58]. These experiments indicate that Rac1 becomes activated in response to the induced activation of Cdc42. Accordingly, we assumed positive regulation of Rac1 by Cdc42 in our model. However, the opposite regulation (from Rac1 to Cdc42) cannot be excluded because bidirectional crosstalk is possible through the interaction of Rac1 and Cdc42 with their common GEFs [10,59].

**Figure 7:**
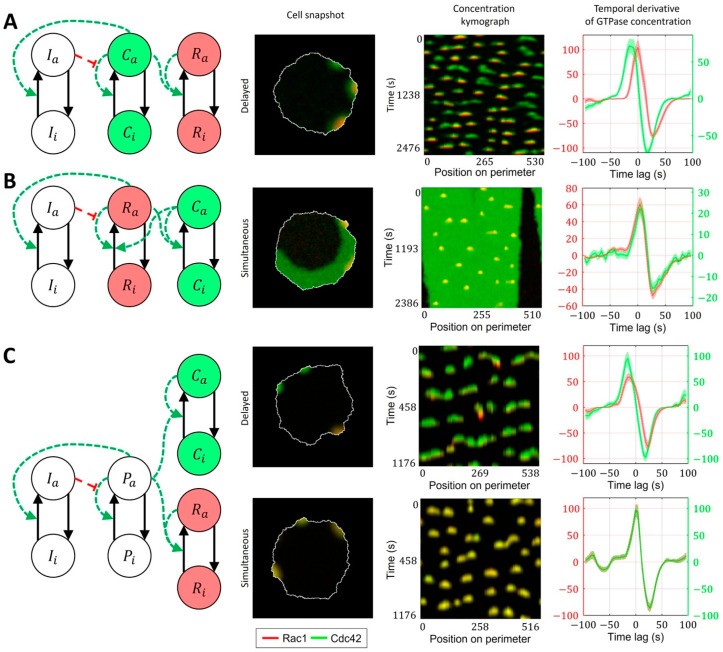
Signaling motifs including both Rac1 and Cdc42. Overlapping regions of Rac1 (red) and Cdc42 (green) activity appear as yellow color. (**A**) A model of Rac1 activation induced by Cdc42 leads to delayed activation of Rac1. (**B**) A bidirectionally coupled model of Cdc42 and Rac1 activation leads to simultaneous activation of GTPases. (**C**) A model of Cdc42 and Rac1 induced by the upstream effector can lead both to simultaneous and delayed activation.

We first considered a model where Rac1 activity is induced by Cdc42 (Figure 7A, Methods, Equations (15)–(20)). Here, the Rac1 component is represented with a bistable MCRD motif. The activation of Rac1 requires a transition from one to the other stable state, which takes place when the active Cdc42 level reaches a certain threshold (Supplementary Figure S3). In this case, Rac1 activation is always delayed relative to the peak of Cdc42 activity. We conclude that such a model is consistent with MEF data but cannot reproduce the simultaneous activation of GTPases in breast cancer cells.

As a next level of complexity, we considered a model where both GTPases are represented with bistable motifs bidirectionally coupled to each other (Figure 7B, Supplementary Video S1). It had been reported that Cdc42 exerts feedback on Rac1 [7,15] and defines cell polarization [60,61], while Rac1 drives protrusion and actin polymerization [36]. Thus, we sought to develop a model that captures the roles of Rac1 and Cdc42. Specifically, we expected Cdc42 in our model to generate polarization patch(es) at the cell edge. This way, through the feedback on Rac1, Cdc42 defines the parts of the cell periphery where the activity of Rac1 can drive the protrusion/retraction cycles. Because of the opposite feedback on Cdc42, Rac1, in turn, affects the variation of Cdc42 activity in protrusions. In this case, the increased activation of Cdc42 does not require the transition to a new stable state, and the Cdc42 concentration instead varies near the level of a high-activity stable state. Such increased activity of Cdc42 above the high activity stable state is possible only if the crosstalk between Cdc42 and Rac1 is relatively weak. Mutual activation of two GTPases works as positive feedback, which can switch the system to a state where both Rac1 and Cdc42 can only be in the active state (Supplementary Figure S4). Such a state represents a polarized state of the cell. Although this model accurately reproduces the typical polarized ruffling dynamics, as observed in breast cancer cells (MDA-MB-231), it only reproduces simultaneous but not the delayed activation of Rac1 and Cdc42. Therefore, the model in this form fits breast cancer cell data but not MEF cell data.

As an alternative to the cell ruffling model that relies on feedback between Cdc42 to Rac1, we considered a model where instead of feedback regulation between Cdc42 and Rac1, both GTPases are activated in response to an upstream stimulus that drives their dynamics (Figure 7C, Supplementary Video S2). Such upstream signaling motifs could work through the PI3K pathway, which was reported as a regulator of cell ruffling [41] and can activate Rac1 and Cdc42 through the interactions of their GEFs with phosphoinositides [16]. This model allowed us to obtain both simultaneous activations of GTPases (when the response of the positive feedback in GTPase activation to the upstream stimulus is the same for Rac1 and Cdc42) and the delayed activation of Rac1 (when the threshold of activation for Rac1 was higher than for Cdc42). In the latter case, the difference in the activation thresholds is modulated by the inflection and max values of the Hill function representing the positive feedback (Figure 7C).

**Figure 8:**
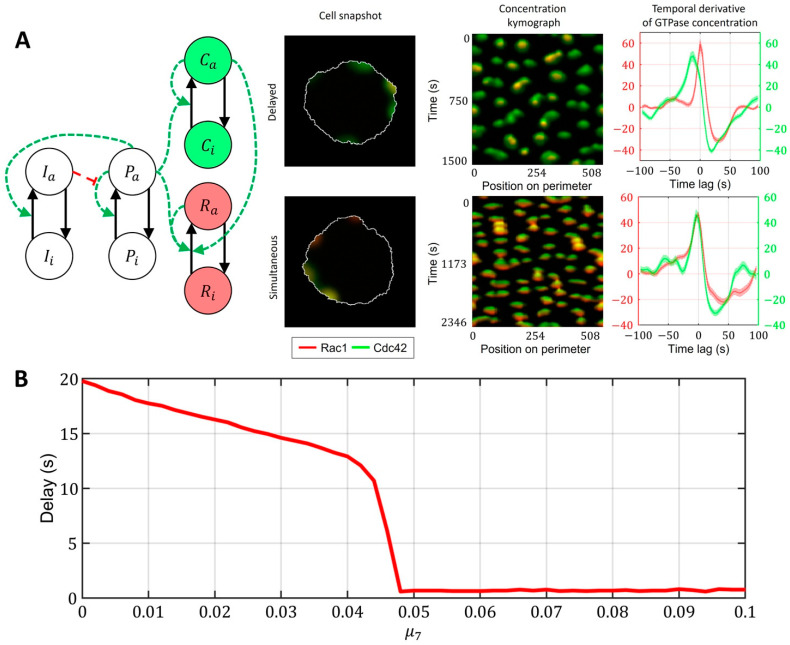
A unified model that accounts for upstream regulator of Cdc42 and Rac1 and for feedback between Cdc42 and Rac1. Overlapping regions of Rac1 (red) and Cdc42 (green) activity appear as yellow color. (**A**) Signaling motif and two cases of dynamics: delayed and simultaneous activation of Rac1 and Cdc42. (**B**) Modulation of feedback between Cdc42 and Rac1 can compensate for the time delay between two GTPases.

Finally, we investigated the role of crosstalk between Cdc42 and Rac1 in the model described in Figure 7C (with the upstream regulator). We found out that even if the responses of Rac1 and Cdc42 to the upstream effector are different and there is a delay in Rac1 and Cdc42 activation (Cdc42 precedes Rac1), the feedback from Cdc42 to Rac1 can compensate for this delay, thereby creating the simultaneous activation of the two GTPases (Figure 8A, Supplementary Video S3). We quantified this effect in the 1D model for various values of the parameter $\mu_{7}$ (Figure 8B). As the strength of the feedback from Cdc42 to Rac1 increases, the delay between Cdc42 and Rac1 becomes negligible. Thus, we conclude that the unified model with the upstream effector motif and the feedback from Cdc42 to Rac1 can explain both the simultaneous dynamics of Cdc42 and Rac1 in the breast cancer cell line (MDA-MB-231) and the delayed activation in the MEF cell line.

## 3. Discussion

Cell morphodynamics is a field encompassing complex, multiscale processes that involve pattern formation and regulation at different stages, from the biochemical regulation of protein activity to the biomechanical regulation of membrane protrusion through the cytoskeleton assembly. Understanding these processes requires an integrative approach that connects multiple levels of regulation and represents them in a holistic manner. Computational modeling can be particularly useful for exploring the underlying mechanisms. By offering virtual experiments, computational modeling can be used to test conceptual biological hypotheses about regulatory mechanisms and provide experimentally testable predictions.

In this study, we built a series of models to capture spatial and temporal GTPase activity and couple it to the protrusion/retraction cycles of cell edge motion along the entire periphery of a cell. We represented protein activity in the form of reaction-diffusion equations, which allowed us to apply the principles of pattern formation in biological morphogenesis to cellular-level dynamics and investigate the spatiotemporal activity of GTPases. The output of our model has the same format as the corresponding experimental imaging data, which enables a direct quantitative comparison of our simulation results and the data.

To perform analyses of the coordination between GTPase activity and cell edge motion, we developed an automated image analysis pipeline that computes a set of trajectories formed by edge motion and tracks both GTPase activity and the edge velocity along these trajectories. We used this pipeline to match GTPase dynamics from experimental FRET biosensor data and from our simulation models. This parallel assessment allowed us to achieve close quantitative agreement of the modeled and observed dynamics of Cdc42 and Rac1 activity during cell ruffling in both breast cancer cells (MDA-MB-231) and MEF cell lines.

Our analysis confirmed that the peaks of Rac1 and Cdc42 activation follow the peak of membrane velocity. Previously, such activity delay was reported by Marston et al. [20,21] and Machacek et al. [20,21]. To explain this phenomenon, we tested two hypotheses about the regulation of protrusive activity. For the first model, we assumed that the presence of active GTPase (e.g., Rac1) is sufficient to maintain the protrusion process; in this case, the cell edge responds to the concentration value of active GTPase. For the second model, we assumed that the increase in GTPase activity is needed to maintain the protrusion process; in this case, the cell edge responds to the kinetic rate of GTPase activation. The results made it clear that the first hypothesis does not agree with the experimental data, whereas the second hypothesis yields close agreement with all metrics. Previously, Yamao et al. [24] applied control theory to investigate the dependence of membrane velocity on Rac1 and Cdc42 activity and reported that the response function has the properties of a differentiator circuit. Indeed, this property is consistent with our second model setup, where the kinetic rate constitutes the temporal derivative of Rho-GTPase activation. One possible interpretation is that our results reflect the fact that GTPase activity is powered by GTP hydrolysis, and thus the protrusion cycle may be synchronous with the local influx of GTP. However, caution should be taken in interpreting the dependence of the edge velocity on the derivative of GTPase concentration. Our simulations with significantly decreased deactivation rate (mimicking constitutive GTPase activity) did not lead to protrusion stalling as could be expected but instead shifted the system dynamics to the formation of stable activity patches resembling the formation of broad lamellipodia. Although lamellipodia formation due to the constitutive activity of GTPase mutants (such as Q61L or G12V) was indeed reported by several studies [62,63,64], there are also reports that the constitutive activity of EGFP-Rac1 Q61L completely suppressed the motility of U87MG cells [65] and that Cdc42Hs expression initially produces fine filopodia in Swiss 3T3 fibroblasts before cells generate lamellipodia [66]. Therefore, further and more direct investigations with a sufficient spatiotemporal resolution are needed to provide confirmation or indicate any contradiction with the rate-of-change regulation suggested by the model based on our specific cell line data.

Another focus of our morphodynamic cell modeling effort was the coordination between Rac1 and Cdc42 activity during cell ruffling. In addition to the breast cancer cell line (MDA-MB-231), where both GTPases are activated simultaneously, we applied our image analysis pipeline to a MEF cell line. Since the delay in the peaks of GTPase activity can be cell-type specific (and our data analysis supports that possibility), we explored different models to test if the delay in activity can be modulated within a unified regulatory network. Specifically, we investigated different network motifs involving feedback between Rac1 and Cdc42 and including an upstream effector acting on both Rac1 and Cdc42. The simulation results revealed that in the presence of the upstream regulator (presumably working through activation of phospholipids via the PI3K pathway), both simultaneous and delayed activations of Rac1 and Cdc42 are possible. In this setup, the feedback from Cdc42 to Rac1 can synchronize the activation of the two GTPases.

An additional argument for the importance of the feedback between Cdc42 to Rac1 is that such feedback allows us to capture polarized ruffling, i.e., a dynamic regime in which only one or several distinct parts of the cell undergo persistent ruffling. Our results provide insights into the regulatory mechanisms of GTPases and their role in cell morphodynamics, which can improve our understanding of the underlying biological processes and explain differences in Rac1 and Cdc42 dynamics in various biological contexts.

## 4. Materials and Methods

### 4.1. Reaction-Diffusion Models of GTPase Signaling

A generic reaction-diffusion formulation of the spatial and temporal activity of Rho-GTPases and their regulators in active and inactive forms can be presented as

(1) $$\frac{\partial X_{a}^{j}}{\partial t}=f_{X_{a}^{j}}+D_{X_{a}^{j}}\frac{\partial^{2}X_{a}^{j}}{\partial x^{2}}+D_{X_{a}^{j}}\frac{\partial^{2}X_{a}^{j}}{\partial y^{2}}$$

(2) $$\frac{\partial X_{i}^{j}}{\partial t}=f_{X_{i}^{j}}+D_{X_{i}^{j}}\frac{\partial^{2}X_{i}^{j}}{\partial x^{2}}+D_{X_{i}^{j}}\frac{\partial^{2}X_{i}^{j}}{\partial y^{2}}$$

where $X_{a}$ and $X_{i}$ are two components representing the concentrations of protein X in its active and inactive forms, respectively; the index j refers to different proteins, D is the diffusion coefficient, and f is the reaction term for each component.

To explain the mechanisms of regulation of Rho-GTPase signaling and to guide our choice of parameter values in the subsequent more complex models, we first investigated the two-component system and its response to the modulation of kinetic parameters. As a minimal 1D mass-conserved RD (MCRD) model of a single GTPase, we adopted published equations [42,67,68]:

(3) $$\frac{\partial G_{a}}{\partial t}=\left(k_{1}+\gamma_{1}\frac{G_{a}^{2}}{K_{1}^{2}+G_{a}^{2}}\right)G_{i}-k_{2}G_{a}+D_{G_{a}}\frac{\partial^{2}G_{a}}{\partial x^{2}}$$

(4) $$\frac{\partial G_{i}}{\partial t}=-\left(k_{1}+\gamma_{1}\frac{G_{a}^{2}}{K_{1}^{2}+G_{a}^{2}}\right)G_{i}+k_{2}G_{a}+D_{G_{i}}\frac{\partial^{2}G_{i}}{\partial x^{2}}$$

Here, $G_{a}$ and $G_{i}$ are the concentrations of active and inactive forms of the GTPase, respectively. The numerical values of the parameters (in arbitrary units) are: $k_{1}=0.005$, $\gamma_{1}\in \left[0,6\right]$, $K_{1}\in \left[0,10\right]$, $k_{2}=0.1$, $G_{t.c.}=1$ (the total GTPase concentration), $D_{G_{a}}=0.001$, $D_{G_{i}}=\frac{0.1}{3}$. As the homogeneous initial conditions, we used the values $G_{a}^{init}=0$, $G_{i}^{init}=1$. As the heterogeneous initial conditions, we used: $G_{a}^{init}=20$ for $x\in \left[0,0.1\right]$, $G_{a}^{init}=0$ for $x\in \left[0.1,4\right]$, $G_{i}^{init}=0.5$ for $x\in \left[0,4\right]$. We also added small-magnitude random noise to the initial conditions to create a perturbation to the unstable homogeneous state: $G_{a}^{init}+\xi$, $G_{i}^{init}-\xi$, $\xi =10^{-6}\cdot |N(0,1)|$, where N(0,1) is a normally distributed random variable with a mean of zero and a standard deviation of one. To obtain the numerical solution of the system in the 1D case, we used the built-in MATLAB finite element solver, *pdepe*, with the size of the simulation domain equal to 4 a.u.

The model was used to explore the properties of Rho-GTPase activation in the phase space of $\gamma_{1}$ and $K_{1}$ parameters and define the regions of monostability, bistability, and Turing-instability (see Results). These results were used to calibrate the values of kinetic parameters in the models with a regulator responsible for switching the dynamics of our Rho-GTPase signaling motif between the regimes where: (1) a stimulus can induce activation, (2) the activation emerges because of the unstable homogeneous state, and (3) the system is monostable and inactive.

In the extended models with regulator (inhibitor I), we began with the setting $k_{1}=0.005$, $\gamma_{1}=2$, $K_{1}\in \left[0,6\right].$ We then manually adjusted the parameters governing the inhibitor dynamics, as well as the GTPase response to the inhibitor, to capture excitable and oscillatory dynamics. In all models, the values of membrane-bound and cytosolic proteins were set as $D_{c}=0.001$ and $D_{m}=\frac{0.1}{3}$, respectively. The value of the $K_{1}$ parameter was set differently at the border of the simulation domain. Based on the experimental evidence [15,54,56,57], we assumed that the GTPase activity remained high at the very edge of the cell. Thus, for the region at the boundary of the simulation domain, we used $K_{1}^{edge}=1.4$, while we used $K_{1}^{basal}=2.1$ for the interior nodes (see Figure 2F).

Solutions of the 2D models were obtained with the forward Euler finite difference scheme with time step Δt=0.001 and spatial grid step Δx=0.02. In the 2D version of the model, we also included a noise term to account for intrinsic noise in signaling networks and stochastic switching of GTPases and their regulators between the active and inactive forms:

(5) $$\frac{\partial X_{a}^{j}}{\partial t}=f_{X_{a}^{j}}+D_{X_{a}^{j}}\frac{\partial^{2}X_{a}^{j}}{\partial x^{2}}+D_{X_{a}^{j}}\frac{\partial^{2}X_{a}^{j}}{\partial y^{2}}+\alpha_{X}\xi_{X}$$

(6) $$\frac{\partial X_{i}^{j}}{\partial t}=f_{X_{i}^{j}}+D_{X_{i}^{j}}\frac{\partial^{2}X_{i}^{j}}{\partial x^{2}}+D_{X_{i}^{j}}\frac{\partial^{2}X_{i}^{j}}{\partial y^{2}}-\alpha_{X}\xi_{X}$$

Here, $\alpha_{X}$ is the noise amplitude, and $\xi_{X}$ is a $N\left(0,1\right)$ random variable. To ensure the positivity of the solution and mass conservation, the sign of the noise term is opposite for active and inactive forms of protein; in addition, we applied other checks at each iteration as described in [47]. To obtain solutions for simulations in arbitrarily shaped domains with no-flux boundary conditions, we used the discrete, five-point stencil representation of the Laplace operator and set fluxes across the edge of the domain to zero. For the details of our implementation, see [47]. For simulations in the static square domain, we used 200 × 200 grid points (corresponding to 4 × 4 a.u. domain size). For simulations of a dynamic cell, the initial shape of the cell was set to be circular. The noise amplitude in all simulations was α = 5. Supplementary Figure S1 offers further details of the analysis of the system dynamics for different noise amplitudes and [47] provides details of the textural analysis. For the derivation of the extended model with inhibitor, see Supplementary Text S1.

### 4.2. Four-Component Model of GTPase Activity during Cell Ruffling

This section describes our extended models with the inhibitor, upstream regulator, and one or multiple Rho-GTPases, based on reaction-diffusion equations. For their implementation, we denoted active and inactive forms of GTPase, Inhibitor, Cdc42, Rac1, and an upstream regulator (presumably acting through phospholipids) with $G_{a}$/$G_{i}$, $I_{a}$/$I_{i}$, $C_{a}$/$C_{i}$, $R_{a}$/$R_{i}$, $P_{a}$/$P_{i}$, respectively:

(7) $$\frac{\partial G_{a}}{\partial t}=\left(k_{1}+\gamma_{1}\frac{G_{a}^{2}}{K_{1}^{2}+\beta_{1}I_{a}^{2}+G_{a}^{2}}\right)G_{i}-k_{2}G_{a}+D_{G_{a}}\frac{\partial^{2}G_{a}}{\partial x^{2}}+D_{G_{a}}\frac{\partial^{2}G_{a}}{\partial y^{2}}+\alpha_{G}\xi_{G}$$

(8) $$\frac{\partial G_{i}}{\partial t}=-\left(k_{1}+\gamma_{1}\frac{G_{a}^{2}}{K_{1}^{2}+\beta_{1}I_{a}^{2}+G_{a}^{2}}\right)G_{i}+k_{2}G_{a}+D_{G_{i}}\frac{\partial^{2}G_{i}}{\partial x^{2}}+D_{G_{i}}\frac{\partial^{2}G_{i}}{\partial y^{2}}-\alpha_{G}\xi_{G}$$

(9) $$\frac{\partial I_{a}}{\partial t}=\left(k_{3}+\gamma_{3}G_{a}^{2}\right)I_{i}-k_{4}I_{a}+D_{I_{a}}\frac{\partial^{2}I_{a}}{\partial x^{2}}+D_{I_{a}}\frac{\partial^{2}I_{a}}{\partial y^{2}}+\alpha_{I}\xi_{I}$$

(10) $$\frac{\partial I_{i}}{\partial t}=-\left(k_{3}+\gamma_{3}G_{a}^{2}\right)I_{i}+k_{4}I_{a}+D_{I_{i}}\frac{\partial^{2}I_{i}}{\partial x^{2}}+D_{I_{i}}\frac{\partial^{2}I_{i}}{\partial y^{2}}-\alpha_{I}\xi_{I}$$

and chose the following parameter values:

Kinetic parameters values: $k_{1}=0.005$, $\gamma_{1}=2$, $K_{1}=1.4$ (oscillatory dynamics), $K_{1}=2.1$ (excitable dynamics), $\beta_{1}=0.5$, $k_{2}=0.1$, $k_{3}=10^{-5}$, $\gamma_{3}=0.2$, $k_{4}=10^{-2}$.

Diffusion coefficients: $D_{G_{a}}=0.001$, $D_{G_{i}}=\frac{0.1}{3}$, $D_{I_{a}}=0.003$, $D_{I_{i}}=\frac{0.1}{3}$.

Total concentrations of the components: $G_{t.c.}=1$, $I_{t.c.}=3$.

Initial conditions: $G_{a}^{init}=0$, $G_{i}^{init}=1$, $I_{a}^{init}=0$, and $I_{i}^{init}=3$.

The 1D version of this model was solved with the MATLAB built-in solver *pdepe* (as described above), with minor perturbations in initial conditions and a simulation duration of $5\times 10^{5}$ a.u.

We developed four variations of this baseline model. The parameter values for the core two-component MCRD motif were chosen using the phase space diagram (Figure 2B), showing how the modulation of the parameter $K_{1}$ by a GTPase effector leads to the transition of the two-component MCRD between the Turing-unstable and monostable regimes. The parameters of the extended models (including effector *I*) were further fine-tuned to obtain an agreement with the experimentally observed activity of Rho-GTPases during cell ruffling.

*Model of Cdc42 Induced Activation of Rac1*

(11) $$\frac{\partial C_{a}}{\partial t}=\left(k_{1}+\gamma_{1}\frac{C_{a}^{2}}{K_{1}^{2}+\beta_{1}I_{a}^{2}+C_{a}^{2}}\right)C_{i}-k_{2}C_{a}+D_{C_{a}}\frac{\partial^{2}C_{a}}{\partial x^{2}}+D_{C_{a}}\frac{\partial^{2}C_{a}}{\partial y^{2}}+\alpha_{C}\xi_{C}$$

(12) $$\frac{\partial C_{i}}{\partial t}=-\left(k_{1}+\gamma_{1}\frac{C_{a}^{2}}{K_{1}^{2}+\beta_{1}I_{a}^{2}+C_{a}^{2}}\right)C_{i}+k_{2}C_{a}+D_{C_{i}}\frac{\partial^{2}C_{i}}{\partial x^{2}}+D_{C_{i}}\frac{\partial^{2}C_{i}}{\partial y^{2}}-\alpha_{C}\xi_{C}$$

(13) $$\frac{\partial I_{a}}{\partial t}=\left(k_{3}+\gamma_{3}C_{a}^{2}\right)I_{i}-k_{4}I_{a}+D_{I_{a}}\frac{\partial^{2}I_{a}}{\partial x^{2}}+D_{I_{a}}\frac{\partial^{2}I_{a}}{\partial y^{2}}+\alpha_{I}\xi_{I}$$

(14) $$\frac{\partial I_{i}}{\partial t}=-\left(k_{3}+\gamma_{3}C_{a}^{2}\right)I_{i}+k_{4}I_{a}+D_{I_{i}}\frac{\partial^{2}I_{i}}{\partial x^{2}}+D_{I_{i}}\frac{\partial^{2}I_{i}}{\partial y^{2}}-\alpha_{I}\xi_{I}$$

(15) $$\frac{\partial R_{a}}{\partial t}=\left(k_{5}+(\gamma_{5}+\alpha_{5}C_{s}^{2})\frac{R_{a}^{2}}{K_{5}^{2}+R_{a}^{2}}\right)R_{i}-k_{6}R_{a}+D_{R_{a}}\frac{\partial^{2}R_{a}}{\partial x^{2}}+D_{R_{a}}\frac{\partial^{2}R_{a}}{\partial y^{2}}+\alpha_{R}\xi_{R}$$

(16) $$\frac{\partial R_{i}}{\partial t}=-\left(k_{5}+(\gamma_{5}+\alpha_{5}C_{s}^{2})\frac{R_{a}^{2}}{K_{5}^{2}+R_{a}^{2}}\right)R_{i}+k_{6}R_{a}+D_{R_{i}}\frac{\partial^{2}R_{i}}{\partial x^{2}}+D_{R_{i}}\frac{\partial^{2}R_{i}}{\partial y^{2}}-\alpha_{R}\xi_{R}$$

Kinetic parameters values: $k_{1}=0.005$, $\gamma_{1}=2$, $K_{1}^{edge}=1.4$, $K_{1}^{basal}=2.1$, $\beta_{1}=0.5$, $k_{2}=0.1$, $k_{3}=10^{-5}$, $\gamma_{3}=0.2$, $k_{4}=10^{-2}$, $k_{5}=0.005$, $\gamma_{5}=0.3$, $\alpha_{5}=0.8$, $K_{5}^{edge}=1.4$, $K_{5}^{basal}=2.1$, $k_{6}=0.1$.

Diffusion coefficients: $D_{C_{a}}=0.001$, $D_{C_{i}}=\frac{0.1}{3}$, $D_{I_{a}}=0.003$, $D_{I_{i}}=\frac{0.1}{3}$, $D_{R_{a}}=0.001$, $D_{R_{i}}=\frac{0.1}{3}$.

Total concentrations of the components: $C_{t.c.}=1$, $I_{t.c.}=3$, $R_{t.c.}=1$.

Initial conditions: $C_{a}^{init}=0$, $C_{i}^{init}=1$, $I_{a}^{init}=1.5$, $I_{i}^{init}=1.5$, $R_{a}^{init}=0$, $R_{i}^{init}=1$.

*Model of Bidirectionally Coupled Cdc42 and Rac1 Activity*

(17) $$\frac{\partial R_{a}}{\partial t}=\left(k_{1}+(\gamma_{1}+\alpha_{1}C_{s}^{2})\frac{R_{a}^{2}}{K_{1}^{2}+\beta_{1}I_{a}^{2}+R_{a}^{2}}\right)R_{i}-k_{2}R_{a}+D_{R_{a}}\frac{\partial^{2}R_{a}}{\partial x^{2}}+D_{R_{a}}\frac{\partial^{2}R_{a}}{\partial y^{2}}+\alpha_{R}\xi_{R}$$

(18) $$\frac{\partial R_{i}}{\partial t}=-\left(k_{1}+(\gamma_{1}+\alpha_{1}C_{s}^{2})\frac{R_{a}^{2}}{K_{1}^{2}+\beta_{1}I_{a}^{2}+R_{a}^{2}}\right)R_{i}+k_{2}R_{a}+D_{R_{i}}\frac{\partial^{2}R_{i}}{\partial x^{2}}+D_{R_{i}}\frac{\partial^{2}R_{i}}{\partial y^{2}}-\alpha_{R}\xi_{R}$$

(19) $$\frac{\partial I_{a}}{\partial t}=\left(k_{3}+\gamma_{3}R_{a}^{2}\right)I_{i}-k_{4}I_{a}+D_{I_{a}}\frac{\partial^{2}I_{a}}{\partial x^{2}}+D_{I_{a}}\frac{\partial^{2}I_{a}}{\partial y^{2}}+\alpha_{I}\xi_{I}$$

(20) $$\frac{\partial I_{i}}{\partial t}=-\left(k_{3}+\gamma_{3}R_{a}^{2}\right)I_{i}+k_{4}I_{a}+D_{I_{i}}\frac{\partial^{2}I_{i}}{\partial x^{2}}+D_{I_{i}}\frac{\partial^{2}I_{i}}{\partial y^{2}}-\alpha_{I}\xi_{I}$$

(21) $$\frac{\partial C_{a}}{\partial t}=\left(k_{5}+(\gamma_{5}+\alpha_{5}R_{s}^{2})\frac{C_{a}^{2}}{K_{5}^{2}+C_{a}^{2}}\right)C_{i}-k_{6}C_{a}+D_{C_{a}}\frac{\partial^{2}C_{a}}{\partial x^{2}}+D_{C_{a}}\frac{\partial^{2}C_{a}}{\partial y^{2}}+\alpha_{C}\xi_{C}$$

(22) $$\frac{\partial C_{i}}{\partial t}=-\left(k_{5}+(\gamma_{5}+\alpha_{5}R_{s}^{2})\frac{C_{a}^{2}}{K_{5}^{2}+C_{a}^{2}}\right)C_{i}+k_{6}C_{a}+D_{C_{i}}\frac{\partial^{2}C_{i}}{\partial x^{2}}+D_{C_{i}}\frac{\partial^{2}C_{i}}{\partial y^{2}}-\alpha_{C}\xi_{C}$$

Kinetic parameters values: $k_{1}=0.005$, $\gamma_{1}=1.5$, $\alpha_{1}=0.85$, $K_{1}^{edge}=1.4$, $K_{1}^{basal}=2.1$, $\beta_{1}=0.5$, $k_{2}=0.1$, $k_{3}=10^{-5}$, $\gamma_{3}=0.2$, $k_{4}=10^{-2}$, $k_{5}=0.07$, $\gamma_{5}=1.5$, $\alpha_{5}=0.1$, $K_{5}^{edge}=0.45$, $K_{5}^{basal}=0.5$, $k_{6}=1.15$.

Diffusion coefficients: $D_{R_{a}}=0.001$, $D_{R_{i}}=\frac{0.1}{3}$, $D_{I_{a}}=0.003$, $D_{I_{i}}=\frac{0.1}{3}$, $D_{C_{a}}=0.001$, $D_{C_{i}}=\frac{0.1}{3}$.

Total concentrations of the components: $C_{t.c.}=1$, $I_{t.c.}=3$, $R_{t.c.}=1$.

Initial conditions: $R_{a}^{init}=0$, $R_{i}^{init}=1$, $I_{a}^{init}=0$, $I_{i}^{init}=3$. For Cdc42 I applied initial stimulus: $C_{a}^{init}=1$, $C_{i}^{init}=0$ (at the point of excitation), $C_{a}^{init}=0$, $C_{i}^{init}=1$ (for all other nodes).

*Model of Cdc42 and Rac1 Activity Regulated by the Upstream Effector*

(23) $$\frac{\partial P_{a}}{\partial t}=\left(k_{1}+\gamma_{1}\frac{P_{a}^{2}}{K_{1}^{2}+\beta_{1}I_{a}^{2}+P_{a}^{2}}\right)P_{i}-k_{2}P_{a}+D_{P_{a}}\frac{\partial^{2}P_{a}}{\partial x^{2}}+D_{P_{a}}\frac{\partial^{2}P_{a}}{\partial y^{2}}+\alpha_{P}\xi_{P}$$

(24) $$\frac{\partial P_{i}}{\partial t}=-\left(k_{1}+\gamma_{1}\frac{P_{a}^{2}}{K_{1}^{2}+\beta_{1}I_{a}^{2}+P_{a}^{2}}\right)P_{i}+k_{2}P_{a}+D_{P_{i}}\frac{\partial^{2}P_{i}}{\partial x^{2}}+D_{P_{i}}\frac{\partial^{2}P_{i}}{\partial y^{2}}-\alpha_{P}\xi_{P}$$

(25) $$\frac{\partial I_{a}}{\partial t}=\left(k_{3}+\gamma_{3}P_{a}^{2}\right)I_{i}-k_{4}I_{a}+D_{I_{a}}\frac{\partial^{2}I_{a}}{\partial x^{2}}+D_{I_{a}}\frac{\partial^{2}I_{a}}{\partial y^{2}}+\alpha_{I}\xi_{I}$$

(26) $$\frac{\partial I_{i}}{\partial t}=-\left(k_{3}+\gamma_{3}P_{a}^{2}\right)I_{i}+k_{4}I_{a}+D_{I_{i}}\frac{\partial^{2}I_{i}}{\partial x^{2}}+D_{I_{i}}\frac{\partial^{2}I_{i}}{\partial y^{2}}-\alpha_{I}\xi_{I}$$

(27) $$\frac{\partial C_{a}}{\partial t}=\left(k_{5}+(\gamma_{5}+\alpha_{5}P_{s}^{2})\frac{C_{a}^{2}}{K_{5}^{2}+C_{a}^{2}}\right)C_{i}-k_{6}C_{a}+D_{C_{a}}\frac{\partial^{2}C_{a}}{\partial x^{2}}+D_{C_{a}}\frac{\partial^{2}C_{a}}{\partial y^{2}}+\alpha_{C}\xi_{C}$$

(28) $$\frac{\partial C_{i}}{\partial t}=-\left(k_{5}+\left(\gamma_{5}+\alpha_{5}P_{s}^{2}\right)\frac{C_{a}^{2}}{K_{5}^{2}+C_{a}^{2}}\right)C_{i}+k_{6}C_{a}+D_{C_{i}}\frac{\partial^{2}C_{i}}{\partial x^{2}}+D_{C_{i}}\frac{\partial^{2}C_{i}}{\partial y^{2}}-\alpha_{C}\xi_{C}$$

(29) $$\frac{\partial R_{a}}{\partial t}=\left(k_{7}+(\gamma_{7}+\alpha_{7}P_{s}^{2})\frac{R_{a}^{2}}{K_{7}^{2}+R_{a}^{2}}\right)R_{i}-k_{8}R_{a}+D_{R_{a}}\frac{\partial^{2}R_{a}}{\partial x^{2}}+D_{R_{a}}\frac{\partial^{2}R_{a}}{\partial y^{2}}+\alpha_{R}\xi_{R}$$

(30) $$\frac{\partial R_{i}}{\partial t}=-\left(k_{7}+\left(\gamma_{7}+\alpha_{7}P_{s}^{2}\right)\frac{R_{a}^{2}}{K_{7}^{2}+R_{a}^{2}}\right)R_{i}+k_{8}R_{a}+D_{R_{i}}\frac{\partial^{2}R_{i}}{\partial x^{2}}+D_{R_{i}}\frac{\partial^{2}R_{i}}{\partial y^{2}}-\alpha_{R}\xi_{R}$$

Kinetic parameters values: $k_{1}=0.005$, $\gamma_{1}=2$, $K_{1}^{edge}=1.4$, $K_{1}^{basal}=2.1$, $\beta_{1}=0.5$, $k_{2}=0.1$, $k_{3}=10^{-5}$, $\gamma_{3}=0.2$, $k_{4}=10^{-2}$, $k_{5}=0.005$, $\gamma_{5}=0.1$, $\alpha_{5}=2$, $K_{5}^{edge}=1.4$, $K_{5}^{basal}=2.1$, $k_{6}=0.1$, $k_{7}=0.005$, $k_{8}=0.1$. Simultaneous Rac1 and Cdc42 activation: $\gamma_{7}=0.1$, $\alpha_{7}=2$, $K_{7}^{edge}=1.4$, $K_{7}^{basal}=2.1$. Delayed Rac1 activation: $\gamma_{7}=0$, $\alpha_{7}=15$, $K_{7}^{edge}=4$, $K_{7}^{basal}=6$. For simultaneous/delayed activation, parameters were adjusted differently to represent the difference in the response of Rac1/Cdc42 to the upstream effector.

Diffusion coefficients: $D_{P_{a}}=0.001$, $D_{P_{i}}=\frac{0.1}{3}$, $D_{I_{a}}=0.003$, $D_{I_{i}}=\frac{0.1}{3}$, $D_{C_{a}}=0.001$, $D_{C_{i}}=\frac{0.1}{3}$, $D_{R_{a}}=0.001$, $D_{R_{i}}=\frac{0.1}{3}$.

Total concentrations of the components: $P_{t.c.}=1$, $I_{t.c.}=3$, $C_{t.c.}=1$, $R_{t.c.}=1$.

Initial conditions: $P_{a}^{init}=0$, $P_{i}^{init}=1$, $I_{a}^{init}=0$, $I_{i}^{init}=3$, $C_{a}^{init}=0$, $C_{i}^{init}=1$, $R_{a}^{init}=0$, $R_{i}^{init}=1$.

*Model of Cdc42 and Rac1 Activity Regulated by the Upstream Effector and the Feedback from Cdc42 to Rac1*

(31) $$\frac{\partial P_{a}}{\partial t}=\left(k_{1}+\gamma_{1}\frac{P_{a}^{2}}{K_{1}^{2}+\beta_{1}I_{a}^{2}+P_{a}^{2}}\right)P_{i}-k_{2}P_{a}+D_{P_{a}}\frac{\partial^{2}P_{a}}{\partial x^{2}}+D_{P_{a}}\frac{\partial^{2}P_{a}}{\partial y^{2}}+\alpha_{P}\xi_{P}$$

(32) $$\frac{\partial P_{i}}{\partial t}=-\left(k_{1}+\gamma_{1}\frac{P_{a}^{2}}{K_{1}^{2}+\beta_{1}I_{a}^{2}+P_{a}^{2}}\right)P_{i}+k_{2}P_{a}+D_{P_{i}}\frac{\partial^{2}P_{i}}{\partial x^{2}}+D_{P_{i}}\frac{\partial^{2}P_{i}}{\partial y^{2}}-\alpha_{P}\xi_{P}$$

(33) $$\frac{\partial I_{a}}{\partial t}=\left(k_{3}+\gamma_{3}P_{a}^{2}\right)I_{i}-k_{4}I_{a}+D_{I_{a}}\frac{\partial^{2}I_{a}}{\partial x^{2}}+D_{I_{a}}\frac{\partial^{2}I_{a}}{\partial y^{2}}+\alpha_{I}\xi_{I}$$

(34) $$\frac{\partial I_{i}}{\partial t}=-\left(k_{3}+\gamma_{3}P_{a}^{2}\right)I_{i}+k_{4}I_{a}+D_{I_{i}}\frac{\partial^{2}I_{i}}{\partial x^{2}}+D_{I_{i}}\frac{\partial^{2}I_{i}}{\partial y^{2}}-\alpha_{I}\xi_{I}$$

(35) $$\frac{\partial C_{a}}{\partial t}=\left(k_{5}+(\gamma_{5}+\alpha_{5}P_{s}^{2})\frac{C_{a}^{2}}{K_{5}^{2}+C_{a}^{2}}\right)C_{i}-k_{6}C_{a}+D_{C_{a}}\frac{\partial^{2}C_{a}}{\partial x^{2}}+D_{C_{a}}\frac{\partial^{2}C_{a}}{\partial y^{2}}+\alpha_{C}\xi_{C}$$

(36) $$\frac{\partial C_{i}}{\partial t}=-\left(k_{5}+\left(\gamma_{5}+\alpha_{5}P_{s}^{2}\right)\frac{C_{a}^{2}}{K_{5}^{2}+C_{a}^{2}}\right)C_{i}+k_{6}C_{a}+D_{C_{i}}\frac{\partial^{2}C_{i}}{\partial x^{2}}+D_{C_{i}}\frac{\partial^{2}C_{i}}{\partial y^{2}}-\alpha_{C}\xi_{C}$$

(37) $$\frac{\partial R_{a}}{\partial t}=\left(k_{7}+(\gamma_{7}+\alpha_{7}P_{s}^{2}+\mu_{7}C_{s}^{2})\frac{R_{a}^{2}}{K_{7}^{2}+R_{a}^{2}}\right)R_{i}-k_{8}R_{a}+D_{R_{a}}\frac{\partial^{2}R_{a}}{\partial x^{2}}+D_{R_{a}}\frac{\partial^{2}R_{a}}{\partial y^{2}}+\alpha_{R}\xi_{R}$$

(38) $$\frac{\partial R_{i}}{\partial t}=-\left(k_{7}+\left(\gamma_{7}+\alpha_{7}P_{s}^{2}+\mu_{7}C_{s}^{2}\right)\frac{R_{a}^{2}}{K_{7}^{2}+R_{a}^{2}}\right)R_{i}+k_{8}R_{a}+D_{R_{i}}\frac{\partial^{2}R_{i}}{\partial x^{2}}+D_{R_{i}}\frac{\partial^{2}R_{i}}{\partial y^{2}}-\alpha_{R}\xi_{R}$$

Kinetic parameters values: $k_{1}=0.005$, $\gamma_{1}=2$, $K_{1}^{edge}=1.4$, $K_{1}^{basal}=2.1$, $\beta_{1}=0.5$, $k_{2}=0.1$, $k_{3}=10^{-5}$, $\gamma_{3}=0.2$, $k_{4}=10^{-2}$, $k_{5}=0.005$, $\gamma_{5}=1$, $\alpha_{5}=2$, $K_{5}^{edge}=1.4$, $K_{5}^{basal}=2.1$, $k_{6}=0.1$, $k_{7}=0.005$, $\gamma_{7}=0.1$, $\alpha_{7}=0.4$, $\mu_{7}=0.06$, $K_{7}^{edge}=1.4$, $K_{7}^{basal}=2.1$, $k_{8}=0.1$.

Diffusion coefficients: $D_{P_{a}}=0.001$, $D_{P_{i}}=\frac{0.1}{3}$, $D_{I_{a}}=0.003$, $D_{I_{i}}=\frac{0.1}{3}$, $D_{C_{a}}=0.001$, $D_{C_{i}}=\frac{0.1}{3}$, $D_{R_{a}}=0.001$, $D_{R_{i}}=\frac{0.1}{3}$.

Total concentrations of the components: $P_{t.c.}=1$, $I_{t.c.}=3$, $C_{t.c.}=1$, $R_{t.c.}=1$.

Initial conditions: $P_{a}^{init}=0$, $P_{i}^{init}=1$, $I_{a}^{init}=0$, $I_{i}^{init}=3$, $C_{a}^{init}=0$, $C_{i}^{init}=1$, $R_{a}^{init}=0$, $R_{i}^{init}=1$.

### 4.3. Computational Method for Coupling Reaction-Diffusion Equations with Cellular Morphodynamics

The rationale and general outline for our modeling approach are provided in the Results section. Here, we describe the technical details of its implementation.

The probabilities of local protrusion and retraction were defined as the multiplication of several contributing factors. The ‘geometry factor’ accounts for the local influence of membrane curvature. It is calculated for each pixel depending on the values of the cell mask in the 8-connected neighborhood of that pixel. The formulas below ensure that the regions with large positive curvature have a lower probability of further protruding and a higher probability of retracting. In contrast, the regions with large negative curvature have a higher probability of protruding and a lower probability of retracting. The geometry factors for protrusion and retraction probabilities are, respectively:

(39) $$w_{p|\;i,j}^{geom}={\left(\sum_{i^{\prime },j^{\prime }\in \left[-1,1\right]}\frac{m_{i+i^{\prime },j+j^{\prime }}}{{\left\|{\vec{r}}_{i,j}-{\vec{r}}_{i+i^{\prime },j+j^{\prime }}\right\|}^{g}}\right)}^{k}$$

(40) $$w_{r|\;i,j}^{geom}={\left(\sum_{i^{\prime },j^{\prime }\in \left[-1,1\right]}\frac{{\overset{-}{m}}_{i+i^{\prime },j+j^{\prime }}}{{\left\|{\vec{r}}_{i,j}-{\vec{r}}_{i+i^{\prime },j+j^{\prime }}\right\|}^{g}}\right)}^{k}$$

where $m_{i,j}$ represents the value of a binary cell mask in position (i,j), ${\overset{-}{m}}_{i,j}$ is the value of the inversed mask (${\overset{-}{m}}_{i,j}=1-m_{i,j}$), $i^{\prime }$ and $j^{\prime }$ are pixel locations in the 8-connected neighborhood of pixel (i,j), $\left\|{\vec{r}}_{i,j}-{\vec{r}}_{i+i^{\prime },j+j^{\prime }}\right\|$ is the Euclidian distance between positions $\left(i,j\right)$ and $\left(i+i^{\prime },j+j^{\prime }\right)$, g defines the sensitivity of the geometry factor to the curvature, and k defines the relative contribution of this factor to the overall probability (i.e., higher values of k lead to a smoother appearance of the cell outline). The indexes ‘*p*’ and ‘*r*’ stand for protrusion and retraction, respectively. In this form, the geometry factor accounts for cell shape in the neighborhood of each boundary pixel. We used a 3 × 3 neighborhood, but the formula can be used in general for a larger neighborhood with the contribution of its pixels decreasing with the distance. For that reason, we included the distance between a pixel $\left(i,j\right)$ and the pixels in its neighborhood as the denominator.

For the volume and actin factors, we have chosen the sigmoidal shape of the probability functions for protrusion and retraction so that these probabilities (and the corresponding cell response) are sensitive to deviations from baseline values of cell volume and actin polymerization rate. Significant deviation from these values corresponds to the terminal values of the probability, zero and one, representing the domination of protrusion over retraction or vice versa.

The ‘volume factor’ controls the conservancy of cell size with a step-like dependence, ensuring that the increase in cell volume decreases the probability of further protrusion and increases the probability of retraction. Correspondingly, the decrease in cell volume has the opposite effect.

(41) $$w_{p\;i,j}^{vol}=\frac{1}{2}+\beta_{V}-\left(\beta_{V}+\gamma_{V}\right)\left(1-\frac{1}{1+e^{\alpha_{V}(V-V_{0})}}\right)$$

(42) $$w_{r\;i,j}^{vol}=\frac{1}{2}-\beta_{V}+\left(\beta_{V}+\gamma_{V}\right)\left(1-\frac{1}{1+e^{\alpha_{V}(V-V_{0})}}\right)$$

Here V and $V_{0}$ represent the current and initial volumes of the cell, and $\alpha_{V}$ regulates the sharpness of the step function. When the volume increases significantly compared to $V_{0}$, $w_{p\;i,j}^{vol}\approx \frac{1}{2}+\gamma_{V}$ and $w_{r\;i,j}^{vol}\approx \frac{1}{2}-\gamma_{V}$, so that $\gamma_{V}$ is the deviation of protrusion and retraction probabilities from the equal 0.5 value. In contrast, when the volume decreases considerably with respect to $V_{0}$, $w_{p\;i,j}^{vol}\approx \frac{1}{2}+\beta_{V}$, and $w_{r\;i,j}^{vol}\approx \frac{1}{2}-\beta_{V}$, so that the deviation of protrusion and retraction probabilities from 0.5 is defined by the value of $\beta_{V}$. Thus, parameters $\beta_{V}$ and $\gamma_{V}$ together define the sensitivity of cell dynamics to the deviation of its volume from a constant value.

The ‘actin factor’ is introduced similarly to the volume factor but with opposite signs in the step-like function to ensure that higher rates of actin polymerization lead to protrusion and lower rates lead to retraction:

(43) $$w_{p\;i,j}^{act}=\frac{1}{2}-\beta_{A}+\left(\beta_{A}+\gamma_{A}\right)\left(1-\frac{1}{1+e^{\alpha_{A}(A-A_{act})}}\right)$$

(44) $$w_{r\;i,j}^{act}=\frac{1}{2}+\beta_{A}-\left(\beta_{A}+\gamma_{A}\right)\left(1-\frac{1}{1+e^{\alpha_{A}(A-A_{act})}}\right)$$

Here, A represents the effects of actin polymerization on the protrusion and retraction probabilities. Such effects can be influenced by the concentration of active GTPase molecules or by the temporal derivative of GTPase activation (the need for this distinction is described in the Results section). $A_{act}$ defines the value of A at the inflection point of the step-like function. In all reported results, we assumed $\beta_{A}=0$, $\gamma_{A}>0$, which implies that weak actin regulation does not create a relative shift in the protrusion and retraction probabilities, while upregulation ($A>A_{act}$) leads to an increased protrusion rate and a decreased retraction rate.

In our models, changes in cell shape result from the addition (protrusion) or removal (retraction) of a pixel at the edge of the cell mask. Such events are determined by calculating the probabilities of protrusion and retraction for each foreground and background pixel along the outline of the cell mask. To calculate the actin factor for a background pixel, we extrapolate the values of concentrations (or kinetic rates) from the RD model by averaging the values in the pixels of the cell mask within the 8-connected neighborhood of this background pixel:

(45) $$\langle A_{i,j}\rangle =\frac{\sum_{i^{\prime },j^{\prime }\in \left[-1,1\right]}A_{i+i^{\prime },j+j\prime }}{\sum_{i^{\prime },j^{\prime }\in \left[-1,1\right]}m_{i+i^{\prime },j+j\prime }}$$

Based on these probabilities, a random number generator selects a set of added and removed pixels. During the update of the cell mask (both for protrusion and retraction steps), we apply an additional automated filtering step that guarantees the unity of the simulation domain, i.e., the algorithm prohibits events that lead to spur (diagonal 4-disconnected) pixels or the formation of hollow or 4-disconnected parts of cell mask. To guarantee mass-conservation of the signaling components, we subtract the total molecular mass in the added pixels evenly from all pixels in the new mask. Because the number of added pixels is always a small fraction of all pixels in the simulation domain, the subtracted value is relatively small. To ensure the positivity of concentrations, we also check that the subtraction is not applied to pixels with concentrations less than the subtracted value. With this approach, the increase in the area of the simulation domain leads to a decrease in the total concentration but not the total mass of the RD components, as it was also implemented in other studies [69]. Similarly, for retraction events, the total molecular mass of the components in the RD model from the removed pixels is evenly distributed across the whole new mask. With this approach, a decrease in the size of the simulation domain leads to an increase in concentration values while the mass is still conserved. After one protrusion and one retraction event, we run a sequence of 50 iterations of the RD model with the forward Euler method in the updated cell mask (as described above). The state of the system was saved after every 1000 iterations of the RD model, which is the 1 a.u. of the simulation time.

The values of parameters associated with geometry and volume factors were the same in all models: g=2, k=3, $\alpha_{V}=10^{-3}$, $V_{0}=\sum_{i,j}m_{i,j}^{0}$, $\beta_{V}=0.5$, $\gamma_{V}=0.5$. For the actin factor, we adjusted parameters separately for each model because of the different kinetic parameters in RD equations. The parameters were adjusted to (1) select the threshold for activation of the actin factor ($A_{act}$); (2) define the sharpness of the sigmoid function ($\alpha_{A}$) that matches the timescale of changes in the concentration of GTPase or the kinetic rate of its activation and (3) match the protrusion rate by adjusting $\gamma_{A}$ parameter.

In all simulations presented in this work, we used the value $\beta_{A}=0$. The values of other parameters related to the actin factor are shown in Table 1:

### 4.4. Image Analysis Pipeline for Coupled Analysis of Cell Edge Velocity and Biosensor Signaling

A conceptual overview of our image analysis pipeline is provided in the Results section. Here, we describe the technical details of its implementation.

As input for our analysis, we used experimental FRET biosensor data with the ratio signal [20,52]. For plotting, we adjusted the gray-scale intensity limits to [$\tilde{I}-6\cdot MAD\left(I\right),\;\tilde{I}+6\cdot MAD\left(I\right)$], where $\tilde{I}$ is the median value of the biosensor signal across the whole time series data and $MAD\left(I\right)$ is a median absolute deviation. We also disregarded outliers in the histogram of the biosensor signal if the number of pixels outside the interval $\left[I-5,\;I+5\right]$ was less than 20. After that, the signal was scaled as $\left(I-\min\left(I\right))/(\max\left(I\right)-\min\left(I\right)\right)$.

Using our mid-contour approach described in the main text, we built a large set of trajectories along which each individual point of the cell outline moves over the course of the whole time-lapse recording. Such trajectories represent local directions of fast protrusion/retraction cycles, while the curving of these trajectories reflects the slow change of the overall cell shape over multiple protrusion/retraction events.

To analyze the biosensor signal in the neighborhood of local edge velocity maxima, we first filtered out trajectories with absolute values of velocity larger than 20 standard deviations (which usually represent cell segmentation artifacts). We identified regions of kymographs with high values of velocity using the criterion: $v>\tilde{v}-2\cdot MAD\left(v\right)$ and filtered out regions of a size smaller than 10 pixels. The points of local velocity maxima were found in the identified regions. The values of cell edge velocity and the biosensor signal were analyzed in the time interval ± 20 frames with respect to the time of the velocity peaks. We then computed the mean values at each time point (along all trajectories) and computed 97.5% confidence intervals. The temporal derivative of the biosensor signal was computed based on the difference from the previous time frame. The coordinate of the membrane was computed as an integral of velocity starting from the first frame in the time interval.

For the analysis of multiplexed Rac1 and Cdc42 data, we processed each GTPase channel separately and combined the results in the time intervals around the time of velocity peaks. Data were averaged over all 6 cells in each dataset.

The same pipeline for the analysis of GTPase concentration was applied to quantify our simulation results. The simulated time series were recorded with a 10 a.u. step.

To match the time and space units between simulations and experimental data, we used temporal and spatial autocorrelation plots computed based on velocity kymographs (Supplementary Figure S2). Based on this analysis, 1 a.u. of simulation time is equal to 0.65 s and 1 a.u. of spatial dimension is equal to 10.127 microns.

## 5. Conclusions

The scientific community may benefit from our image analysis and modeling platforms in future studies of the mechanisms of cell motility driven by spatial and temporal dynamics of the regulatory proteins. In fact, our computational framework can be used as a platform to study other types of cellular morphodynamics and their regulation by signaling pathways. Because the output of the model matches the discretization and resolution of experimental images, it is straightforward to compare simulation results with microscopy data using the same methods of analysis. We envision that by reaching a quantitative agreement between the data and our simulations of the complex interplay between GTPase activity and cell edge dynamics, this work will stimulate further development of integrative models to study the multiscale regulation of morphodynamics in different cell types and under different conditions.

## Figures and Tables

**Table 1:** Parameter choices for the different cell morphodynamic models. The other parameters were the same for all models and provided in the text of the Method section.

Model	$\alpha_{A}$	$A_{act}$	$\gamma_{A}$
GTPase activity in cell ruffling (regulation by absolute concentration value)	30	0.7	0.1
GTPase activity in cell ruffling (regulation by activation rate)	50	0.1	0.2
Rac1 activation by Cdc42	50	0.3	0.2
Coupled model with bidirectional feedback between Rac1 and Cdc42	50	0.2	0.35
Rac1 and Cdc42 activation by the upstream regulator (simultaneous activation)	100	0.1	0.1
Rac1 and Cdc42 activation by the upstream regulator (delayed activation)	30	0.5	0.1
Rac1 and Cdc42 activation by the upstream regulator with feedback between Cdc42 and Rac1 (simultaneous and delayed activation)	30	0.3	0.1

## Data Availability

MATLAB code for modeling and data analysis that was used in this study is available in the following GitHub repositories: https://github.com/tsygankov-lab/Coupled_RDE-Morphodynamics_Model (accessed on 14 June 2023); https://github.com/tsygankov-lab/Cell_Ruffling_Quantification (accessed on 14 June 2023).

## Supplementary Materials

The following supporting information can be downloaded at: https://www.mdpi.com/article/10.3390/cells12121638/s1, 1. Supplementary Information as a single PDF file. 2. Supplementary Videos S1–S3. Video S1. An example of a simulation output for the model of cell ruffling with a bidirectional coupling of Cdc42 and Rac1 activity. Cdc42 defines cell polarization (green color) and the location where it induces Rac1 activity. Rac1 (red color) forms transient activity bursts, activating Cdc42 above the level in the polarization patch and driving cell protrusion. Video S2. An example of a simulation output for the model of cell ruffling where the external regulator induces Cdc42 (green) and Rac1 (red) activities. The movie shows two cases: delayed and simultaneous activation of Rac1 and Cdc42 due to a difference in the parameters of the response to the external regulator. Video S3. An example of a simulation output for the model of cell ruffling where Cdc42 (green) and Rac1 (red) activities are induced by the external regulator (as in Supplemental Video S2) but with additional feedback from Cdc42 (green) to Rac1 (red). The movie shows two cases: delayed and simultaneous activation of Rac1 and Cdc42 due to a difference in the strength of the feedback from Cdc42 to Rac1.
